# Supplementary material for: Transcriptome analysis based on RNA-seq of common innate immune responses of flounder cells to IHNV, VHSV, and HIRRV
Source: PLoS One. 2020 Sep 28;15(9):e0239925. doi: 10.1371/journal.pone.0239925 (PMC7521715; doi:10.1371/journal.pone.0239925)
Supplement: Attachment — Submitted filename: Response to reviewers-JW Park.docx [file pone.0239925.s007.docx]

Dr. Maria del Mar Ortega-Villaizan
Academic Editor
PLOS ONE

Dear Dr. Maria del Mar Ortega-Villaizan,

I am sending a revised version of my manuscript, PONE-D-20-17834. The comments of the reviewers were very helpful to me. So I could improve my manuscript by modification according to the comments. In addition, I prepared responses to each point raised by editor and reviewers and enclose them with this letter.

I hope everything is going well for you.

Sincerely yours

Jeong Woo Park

Department of Biological Sciences

University of Ulsan

Ulsan 44610, Korea

E-mail: jwpark@ulsan.ac.kr

**Journal requirements:**

1. Please ensure that your manuscript meets PLOS ONE's style requirements, including those for file naming. The PLOS ONE style templates can be found at https://journals.plos.org/plosone/s/file?id=wjVg/PLOSOne_formatting_sample_main_body.pdf and <https://journals.plos.org/plosone/s/file?id=ba62/PLOSOne_formatting_sample_title_authors_affiliations.pdf>

***Response***: We modified our manuscript to meet PLOS ONE’s style requirements.

2. In your Methods section, please provide additional details regarding the cell lines used in your study and ensure you have described the source.

For more information regarding PLOS' policy on materials sharing and reporting, see

https://journals.plos.org/plosone/s/materials-and-software-sharing#loc-sharing-materials

and for more information on PLOS ONE's guidelines for research using cell lines, see

https://journals.plos.org/plosone/s/submission-guidelines#loc-cell-lines

***Response***: According to PLOS’ policy and PLOS ONE’s guidelines, we provided additional details regarding two cell lines HINAE and EPC used in this study.

**Reviewers' comments:**

1. Is the manuscript technically sound, and do the data support the conclusions?
The manuscript must describe a technically sound piece of scientific research with data that supports the conclusions. Experiments must have been conducted rigorously, with appropriate controls, replication, and sample sizes. The conclusions must be drawn appropriately based on the data presented.

Reviewer #1: Yes

Reviewer #2: Yes

2. Has the statistical analysis been performed appropriately and rigorously?

Reviewer #1: Yes

Reviewer #2: I Don't Know

***Response***: We performed the statistical analysis wherever required and indicated the program used for statistical analysis at the Figure legend.

3. Have the authors made all data underlying the findings in their manuscript fully available?

The [PLOS Data policy](http://www.plosone.org/static/policies.action#sharing) requires authors to make all data underlying the findings described in their manuscript fully available without restriction, with rare exception (please refer to the Data Availability Statement in the manuscript PDF file). The data should be provided as part of the manuscript or its supporting information, or deposited to a public repository. For example, in addition to summary statistics, the data points behind means, medians and variance measures should be available. If there are restrictions on publicly sharing data—e.g. participant privacy or use of data from a third party—those must be specified.

Reviewer #1: Yes

Reviewer #2: No

***Response***: In original manuscript, we provided raw data underlying the finding described in the manuscript as 4 supplementary Tables. According to the reviewer’s comment, we added two more supplementary Table (S4 and S6 Tables) and provided information about the source of PCR primer in Table 3.

4. Is the manuscript presented in an intelligible fashion and written in standard English?

PLOS ONE does not copyedit accepted manuscripts, so the language in submitted articles must be clear, correct, and unambiguous. Any typographical or grammatical errors should be corrected at revision, so please note any specific errors here.

Reviewer #1: Yes

Reviewer #2: No

***Response***: According to the reviewer’s comments, we modified our manuscript by reducing ambiguity in several parts, correcting errors, and providing detail information.

5. Review Comments to the Author

**Reviewer #1**:

General comments
This paper presents a comparative study on three fish rhabdoviruses (IHNV, VHSV and HIRRV) infection of a flounder cell line. It is noteworthy that while VHSV and HIRRV are pathogenic to flounder, IHNV is not. However, the three viruses replicate efficiently in flounder cells. Transcriptomic/RNAseq analysis revealed some pathways and immune-related genes modulated by the viral infections. The three viruses share a number of DEGs in common, while there are many pathways that only show up in one of the viral infections. Overall, this work contributes with novel information to the field of fish virology, but in the end the authors seemed unable to provide an explanation on why IHNV does not cause disease to flounder. It is somehow unfortunate that the key question regarding the difference between the two pathogenic viruses (VHSV and HIRRV) and the non-pathogenic IHNV remains unanswered. The suggestion that IHNV cannot evade the cell antiviral response is not convincing, since there is no hard evidence supporting this hypothesis. In fact, this paper shows that IHNV replication on flounder cells does not differ from the other two rhabdoviruses. It is likely that some factors involved in this phenomenon are missed in the cell culture approach. A follow up work in vivo would surely shed more light on this interesting topic.

***Response***: According to your comment, we deleted the sentence “Thus, it is likely that, while VHSV and HIRRV can evade adaptive immune responses of flounder, IHNV may be sensitive to them.” from the Discussion section. Regarding *in vivo* experiment, we are now conducting RNA-seq analysis using tissues obtained from flounder infected with VHSV, HIRRV, and IHNV to identify genes or factors which may be responsible for the different pathogenicity among them.

Specific issues
Materials and methods. All three viruses were grown at 20ºC. Although IHNV can grow at 20ºC, such temperature is higher than the reported optimal replication temperature for IHNV, which is around 14ºC. Nevertheless, here efficient replication of IHNV at 20ºC is shown, so I guess it is alright.

***Response***: As you indicate, the optimum temperature for IHNV is around 14ºC. However, since the purpose of this study is to compare transcriptome of host cells infected by three rhabdoviruses, we cultivated cells infected by three rhabdoviruses at the same temperature 20ºC.

One striking piece of data here is that VHSV affects many more (more than twice) immune-related genes than IHNV or HIRRV. This is a remarkable finding, not discussed in the paper. I believe that author should comment on this subject.

***Response***: According to your comment, we added more discussion on the data showing many more immune-related genes in VHSV-infected cells than those in IHNV- or HIRRV-infected cells.

P18. “there is no report on the role of CH25H in virus infected fish cells” I don´t think this is correct. The authors may want to take a look at a couple of papers (1) Pereiro, P., et al. (2017). "Interferon-independent antiviral activity of 25-hydroxycholesterol in a teleost fish." Antiviral Res 145: 146-159. (2) Zhang, Y., et al. (2019). "Fish cholesterol 25-hydroxylase inhibits virus replication via regulating interferon immune response or affecting virus entry." Front. Immunol 10(doi: 10.3389/fimmu.2019.00322).

***Response***: According to your comment, we added information in the “Discussion” section about the role of CH25H in protection of fish cells from fish virus infection with two additional citations.

Generally speaking, data from “omics” techniques are not easy to fit into a concrete mechanistic model. In this sense, I understand that the two pathogenic viruses (VHSV, HIRRV) should have some degree of similarity, while the transcriptome of IHNV-infected cells would differ in a number of genes/pathways that would have a connection to viral pathogenesis. So perhaps the answer is in those genes with a>2-fold decrease only in IHNV-infected cells. (which are hardly mentioned in the discussion).

***Response***: As you indicated, two groups of genes would have a connection to novirhabdoviral pathogenesis: one group of genes which are commonly modulated by both VHSV and HIRRV but not by IHNV; the other group of genes which are regulated only by IHNV. According to your comment, we mentioned those genes with possible connection to viral pathogenesis in the “Discussion” section.

**Reviewer #2:**

The manuscript title “Transcriptome analysis based on RNA-seq of common innate immune responses of flounder cells to IHNV, VHSV, and HIRRV “by Kwang Il Kim and colleagues presents a work about the study of the transcriptomic profile of HINAE cells infected with IHNV, VHSV and VHSV. The results indicate that innate immune response genes were commonly modulated by all three novirhabdoviruses.
Despite the interesting study, it is necessary to do deep corrections in order to be published. In addition, the manuscript needs a profound English correction. I have divided the correction in sections:

In Material and methods
- Indicate in material and methods the specie (Paralichthys olivaceus) in HIRAME cells.

***Response***: According to your comment, we added the information of the source of the HINAE, olive flounder (*Paralichthys olivaceus*), in “Materials and methods” with a reference.

-The name of EPC cells has to be change to fathead minnow epithelioma papulosum cyprinid as indicate in the paper Winton J, Batts W, deKinkelin P, LeBerre M, Bremont M, Fijan N.Current lineages of the epithelioma papulosum cyprini (EPC) cell line are contaminated with fathead minnow, Pimephales promelas, cells. J Fish Dis.2010) 33:701–4. doi: 10.1111/j.1365-2761.2010.01165.x

***Response***: According to your comment, we added information about the source of EPC as fathead minnow (*Pimephales promelas*) in “Materials and methods” with a reference (Winton et al., 2010) you indicated.

-Authors should indicate in material and methods that “h “means hours at least the first time it appears.

***Response***: According to your comment, we indicated that “h” means hour at the first time it appears in “Materials and methods”.

- I found diverse style in the way to name the commercial references in material and methods, for example some with only the name of the company and others with the name and place please unify them. In addition, you should revise them because I found some reactives products without the commercial reference such as fetal calf serum. Please correct them.

***Response***: According to your comment, we unify the way to name the commercial references in “Materials and methods”. In addition, we added the commercial references to all reagents in the “Materials and methods”.

-Which analysis method do you used to calculate the expression of genes in the real time PCR? Maybe 2^-DDCT?. You should indicate it in material and methods.

***Response***: As you indicate, we used 2^-ΔΔCt^ method to calculate the expression of genes in the real-time PCR. According to your comment, we indicated it in “Materials and methods” with a reference.

-Indicate the name and reference of statistical programme used to do the analysis and also the programm where graphs were performs.

***Response***: According to your comment, we added the name and reference of statistical programme used to do the analysis. We also added the name of the programme in the figure legends where it was used in the graph.

In the results figures and tables

-In section 3.1 I found the next sentence difficult to understand “At day 1 (24 h) p.i., although the titer of IHNV was 6.3 and 6.9-fold lower for 1 and 0.1 MOI than that of VHSV, it was 1.6 and 2.1-fold higher for 1 and 0.1 MOI than that of HIRRV (Fig. 1)”. Please rewrite the sentence.

***Response***: According to your comment, we rewrote the sentence to make it easy to understand as follow: “At day 1 (24 h) p.i., although the titer of IHNV was 6.3~6.9-fold lower than that of VHSV, it was 1.6~2.1-fold higher than that of HIRRV (Fig. 1)”

-In section 3.2 indicate the reference of GSEA programme.

***Response***: according to your comment, we added the reference of GSEA programme.

-In the section 3.4 the genes,” IRF8, CXCR1, TIRAP, CH25H, CXCL11.5, and TLR2, were increased; 3 genes, CCR6a, IL12a, and TLR1” should include the full name of the genes in the text because it is the first time that these genes appear in the manuscript. Please check all the manuscript in case there were another mistake.

***Response***: According to your comment, we provided the full name of these 9 genes in the text. In addition to the 9 genes, we added full name of other genes where they first appeared in the text.

-In the figure 1, the legend indicates that “viral titers were determined in duplicate” the mean and standard deviation should be included in the graph results and indicate in the legend. In addition, the quality of the image is not good; the titles of the X and Y axes are a little bit blurry. Please, improve the image.

***Response***: According to your comment, we added the mean and standard deviation in the Fig. 1 results. In addition, we replaced the result with an improved image.

-In figure 2 the graph of HIRRV should have the same scale as VHSV and IHNV. Please correct it. In addition, it is really surprising that all the graphs are almost identical. I try to check the raw data in supplementary table S1,2 and3 however it is difficult to see the genes that belong to each pathway, can you do another table where only appear the raw data of the graphs ?.

***Response***: According to your comment, we corrected the Fig 2 graph to have the same Y scale. In addition, we provided the raw data of the Fig. 2 graphs as S4 Table.

- In figure 3 A, the graph represents a huge amount of genes so it is impossible to identify each gene because the title is so tiny and it is no possible to read. To solve the problem I do not know if increasing the size of the graph could improve the problem. Maybe the solution is only representing a few important data in the graph. Moreover, the reference of Venn diagrams should be included in the legend.

***Response***: According to your comment, we prepared a new graph based on 23 innate immune response genes with more than 4-fold change in expression by IHNV, VHSV, or HIRRV infection and added this as Fig. 3B. In addition, we provided the reference of Venn diagram in the Figure legend.

-In figure 4 is represented the expression of genes at 12 and 24 hours, but in the results there is not commented the 12 hours data. In my opinion these data should be commented or removed from the graphs. Moreover, I think it is important to have a supplementary table with the Ct data from real time PCR.

***Response***: According to your comment, we deleted the 12 h data from the Fig. 4. In addition, we provided Ct data from the real time PCR as S6 Table.

-In figure 5C and D, some circles cover the nodes of the image. Please correct them.

***Response***: According to your comment, we corrected the circles in the Fig. 5C and 5D not to cover the nodes of the image.

-In table 2: there is not reference or accession number of the primers, please add them.

***Response***: According to your comment, we added accession number of the primers. In addition, we added the information about primers for three genes, CCR6A, IL-12A, and TLR1 which were missed in the manuscript.

- In table 4, I am not sure if the symbol “ # of genes” is a mistake. In the case that it was a mistake, please correct it.

***Response***: The symbol “#” represent “number”. To make it clear, we replaced “#” with “No.”.

- Table 5 should include in the legend the full name of every gene mentioned in the table.

***Response***: According to your comment, we added the full name of every gene in the legend.

In the discussion

The discussion needs a deep review. I miss a more argued discussion in general. For example, I miss in the discussion a comparative between these transcriptomic results with other transcriptomic analysis with other fish infected with VHSV, IHNV and a microarray analysis with HIRRV.

***Response***: According to your comment, we added a comparative between our results with other transcriptomic analysis in the “Discussion” section.

Apart from that , I found the next sentences:
-This sentence “In addition, unigenes could be mapped to 55 KEGG pathways, indicating that novirhabdovirus infection modulates the expression of cellular genes involved in a broad variety of pathway”.it should be included in the results.

***Response***: According to your comment, we included this sentence in the “Results” section.

-“As in IRF8, the other genes in the group of six that were up-regulated, CXCR1, TIRAP, CH25H, CXCL11.5, and TLR2, have been reported to be induced by virus infection and have both antiviral and proviral functions”. This sentence should include a reference.

***Response***: According to your comment, we added references for that sentence.

-“Binding of CXCL8/IL-8 to CXCR1 induces inflammation by recruiting immune cells to the site of damage and infection [60] and co-injection of IL-8 with VHSV G-encoding DNA vaccine in rainbow trout enhanced the production of proinflammatory cytokine [61]. This suggests the possibility that CXCL8/IL-8 induced by virus may enhance antiviral immunity. However, there are several reports indicating that CXCL8/IL-8 inhibits the antiviral immunity of the host. IL-8 induced by virus infection inhibits IFN-α activity in a dose-dependent manner [62] and IL-8 enhances the replication of several viruses including CMV [63], encephalomyocarditis virus (EMCV), and poliovirus [62]”. This paragraph should be better explain, it is confusing.

***Response***: According to your comment, we modified that paragraph to make it clear.

6. PLOS authors have the option to publish the peer review history of their article ([what does this mean?](https://journals.plos.org/plosone/s/editorial-and-peer-review-process#loc-peer-review-history)). If published, this will include your full peer review and any attached files.


If you choose “no”, your identity will remain anonymous but your review may still be made public.


**Do you want your identity to be public for this peer review?** For information about this choice, including consent withdrawal, please see our [Privacy Policy](https://www.plos.org/privacy-policy).

Reviewer #1: No

Reviewer #2: No
