# Supplementary material for: Transcriptome analysis based on RNA-seq of common innate immune responses of flounder cells to IHNV, VHSV, and HIRRV
Source: PLoS One. 2020 Sep 28;15(9):e0239925. doi: 10.1371/journal.pone.0239925 (PMC7521715; doi:10.1371/journal.pone.0239925)
Supplement: Attachment — Submitted filename: Response to reviewers-JW Park.docx [file pone.0239925.s008.docx]

Dr. Maria del Mar Ortega-Villaizan
Academic Editor
PLOS ONE

Dear Dr. Maria del Mar Ortega-Villaizan,

I am sending a revised version of my manuscript, PONE-D-20-17834R1. The comments of the reviewers were very helpful to me. So I could improve my manuscript by modification according to the comments. In addition, I prepared responses to each point raised by reviewer #2 and enclose them with this letter.

I hope everything is going well for you.

Sincerely yours

Jeong Woo Park

Department of Biological Sciences

University of Ulsan

Ulsan 44610, Korea

E-mail: jwpark@ulsan.ac.kr

- In general, the quality of the image of all the figures should be improved. They are blurred and this is the second time that I asked for an improvement in the figures.

***Response***: According to your comment, we improved the quality of images of Figures. Especially, we tried to improve the quality of Fig. 3 and Fig. 5.

- Legend figure1 should indicate that the mean and standard deviation was performed and also indicate the number of samples used in the experiment.

***Response***: According to your comment, we indicated that the mean and standard deviation was performed and, also, we indicated the number of samples used in this experiment in Fig. 1.

- In figure 1 the size of the titles of X and Y axes are not the same please correct them.

***Response***: According to your comment, we corrected the size of X and Y title of Fig 1.

- In figure 2 the title of IHNV is in gray and VHSV and HIRRV are in black. Please unifies the title color.

***Response***: according to your comment, we unified the title color in Fig 2.

- In the discussion authors have added a new sentence “In this context, two groups of genes would play a critical role for connection to novirhabdovirus pathogenesis: 5 genes (3 genes up-regulated;2 genes down-regulated) which are commonly modulated more than two-fold by pathogenic both VHSV and HIRRV but not by IHNV; 6 genes which are down-regulated more than twofold only by IHNV”. It is difficult to know which genes are up or down regulated so I think you should identify these genes in order to clarify this sentence.

***Response***: according to your comment, we added the gene names to this sentence.
